# An inequality for generalized complete elliptic integral

**DOI:** 10.1186/s13660-017-1578-6

**Published:** 2017-12-08

**Authors:** Li Yin, Li-Guo Huang, Yong-Li Wang, Xiu-Li Lin

**Affiliations:** 1 Department of Mathematics, Binzhou University, Binzhou City, Shandong Province 256603 China; 2 College of Mathematics and Systems Science, Shandong University of Science and Technology, Qingdao, 266590 China; 3 College of Mathematics Science, Qufu Normal University, Qufu City, Shandong Province 273165 China

**Keywords:** 33E05, generalized complete elliptic integrals, psi function, hypergeometric function, inequality

## Abstract

In this paper, we show an elegant inequality involving the ratio of generalized complete elliptic integrals of the first kind and generalize an interesting result of Alzer.

## Introduction

The generalized complete elliptic integral of the first kind is defined for $r\in(0,1)$ by

$$ K_{p} (r) = \int_{0}^{\frac{\pi_{p}}{2}} \frac{d\theta}{(1-r^{p}\sin _{p}^{p}\theta)^{1-\frac{1}{p}}}= \int_{0}^{1}\frac{dt}{(1-t^{p})^{\frac {1}{p}}(1-r^{p}t^{p})^{1-\frac{1}{p}}}, $$

where $\sin_{p}\theta$ is the generalized trigonometric function and

$$\pi_{p}=2 \int_{0}^{1}\frac{dt}{(1-t^{p})^{\frac{1}{p}}}=\frac{2}{p}B \biggl(\frac{1}{p}, 1-\frac{1}{p} \biggr). $$

The function $\sin_{p}\theta$ and the number $\pi_{p}$ play important roles in expressing the solutions of inhomogeneous eigenvalue problem of *p*-Laplacian $-(|u'|^{p-2}u')'=\lambda|u|^{p-2}u$ with a boundary condition. These functions have some applications in the quasi-conformal theory, geometric function theory and the theory of Ramanujan modular equation. Báricz [1] established some Turán type inequalities for a Gauss hypergeometric function and for a generalized complete elliptic integral and showed a sharp bound for the generalized complete elliptic integral of the first kind in 2007. In 2012, Bhayo and Vuorinen [2] dealt with generalized elliptic integrals and generalized modular functions. Several new inequalities are given for these and related functions.

For more details on monotonicity, inequalities and convexity and concavity of these functions, the reader may refer to [3–5] and [6] and the references therein.

In 1990, Anderson et al. [7] presented the following inequality:

1.1 $$ \frac{K(r)}{K(\sqrt{r})} > \frac{1}{1+r}\quad \mbox{for } r\in(0,1). $$

Inspired by this work, Alzer and Richards [6] gave the refinement of (1.1): for all $r\in(0,1)$, the following inequality

1.2 $$ \frac{K(r)}{K(\sqrt{r})} > \frac{1}{1+\frac{r}{4}} $$

holds true.

It is natural how inequality (1.2) is generalized to $K_{p} (r)$. Our main result reads as follows.

### Theorem 1.1

*For* $r\in(0,1)$ *and* $p\in[1,2]$, *we have*

1.3 $$ \frac{1}{1+\lambda_{p}r} < \frac{K_{p}(r)}{K_{p}(\sqrt{r})} < \frac{1}{1+u_{p}r}, $$

*where the constants* $\lambda_{p}=\frac{1}{p}(1-\frac{1}{p})$ *and* $u_{p}=0$ *are the best possible*.

## Lemmas

### Lemma 2.1

*The function* $\Delta(x)=\frac{1+ax}{1+bx}$ ($1+bx\neq0$) *is strictly increasing* (*decreasing*) *in* $(0, \infty)$ *if and only if* $a-b>0$ ($a-b<0$).

### Proof

Simple computation yields

$$\frac{d}{dx} \biggl(\frac{1+ax}{1+bx} \biggr)=\frac{a-b}{(1+bx)^{2}}. $$

The proof is complete. □

### Lemma 2.2

(Lemma 2.1 in [8])

*The psi function* $\psi(x)=\frac{\Gamma'(x)}{\Gamma(x)}$ *is strictly concave on* $(0,\infty)$ *and satisfies the duplication formula*

2.1 $$ \psi(2x)=\frac{1}{2}\psi(x)+\frac{1}{2}\psi\biggl(x+ \frac{1}{2}\biggr)+\log2 $$

*for* $x>0$.

### Lemma 2.3

(Lemma 3 in [9])

*For* $x>0$, *we have*

2.2 $$ \ln x-\frac{1}{x} < \psi(x) < \ln x -\frac{1}{2x}. $$

### Lemma 2.4

*For* $x>0$ *and* $p\in[1,2]$, *we have*

2.3 $$ \psi\biggl(x+\frac{1}{p}\biggr)+\psi\biggl(x+1- \frac{1}{p}\biggr) > \psi\biggl(x+\frac {1}{2}-\frac{1}{2p} \biggr)+\psi\biggl(x+\frac{1}{2}+\frac{1}{2p}\biggr). $$

### Proof

Using Lemma 2.3, we only need to prove the following inequality:

$$\ln \biggl[\frac{(x+\frac{1}{p})(x+1-\frac{1}{p})}{(x+\frac {1}{2}-\frac{1}{2p})(x+\frac{1}{2}+\frac{1}{2p})} \biggr]+\frac {1}{x+1-\frac{1}{p}}-\frac{1}{x+\frac{1}{p}}>0. $$

For $p\in[1,2]$, we easily obtain $\frac{1}{p}\geq1-\frac{1}{p}$. So, we have

$$\frac{1}{x+1-\frac{1}{p}}-\frac{1}{x-\frac{1}{p}}>0. $$

On the other hand,

$$\begin{aligned}& \frac{(x+\frac{1}{p})(x+1-\frac{1}{p})}{(x+\frac{1}{2}-\frac {1}{2p})(x+\frac{1}{2}+\frac{1}{2p})} \geqslant1 \\& \quad \Leftrightarrow\quad \frac{1}{p} \biggl(1-\frac{1}{p} \biggr) \geqslant \frac{1}{4} \biggl(1-\frac{1}{p} \biggr) \biggl(1+ \frac{1}{p} \biggr) \\& \quad \Leftrightarrow\quad 3 \biggl(\frac{1}{p} \biggr)^{2}-4 \frac{1}{p}+1 \leqslant0. \end{aligned}$$

So, we complete the proof. □

### Lemma 2.5

*We have*

2.4 $$ \lim_{r\rightarrow1}\frac{K_{p}(r)}{K_{p}(\sqrt[p]{r})}=1. $$

### Proof

Applying the asymptotic formula ([10], equality (2))

2.5 $$ F(a,b;a+b;x)\sim-\frac{1}{B(a,b)}\log(1-x) \quad (x\rightarrow1) $$

and expression [10]

2.6 $$ {K_{p}(r)}=\frac{\pi_{p}}{2}F \biggl( \frac{1}{p},1-\frac {1}{p};1,r^{p} \biggr), $$

where $F(a;b;c;z)$ and $B(x,y)$ denote a classical hypergeometric function and a beta function, respectively, we obtain

2.7 $$ \lim_{r\rightarrow1}\frac{F(a,b;a+b;r^{p})}{F(a,b;a+b;r)}=1. $$

Putting $a=\frac{1}{p}$ and $b=1-\frac{1}{p}$, we complete the proof. □

## Proof of Theorem 1.1

Define

$$f_{p}(r)=\frac{2}{\pi_{p}} \biggl[1+\frac{1}{p} \biggl(1- \frac {1}{p} \biggr)r \biggr] K_{p}(r) $$

and

$$g_{p}(r)=\frac{2}{\pi_{p}} K_{p}\bigl(\sqrt[p]{r}\bigr). $$

By applying (2.6), we get

$$f_{p}(r)=\sum_{n=0}^{\infty}(1+ \lambda_{p} r)r^{pn} $$

and

$$g_{p}(r)=\sum_{n=0}^{\infty}(a_{2n}+a_{2n+1} r)r^{2n}, $$

where $a_{n}=\frac{(\frac{1}{p})_{n}(1-\frac{1}{p})_{n}}{(n!)^{2}}$ and $(r)_{n}=r(r+1)\cdots(r+n-1)$.

Because of $1\leqslant p\leqslant2$, we have

$$\frac{f_{p}(r)}{g_{p}(r)}\geqslant\frac{\sum_{n=0}^{\infty}(1+\lambda _{p} r)a_{n} r^{pn}}{\sum_{n=0}^{\infty}(a_{2n}+a_{2n+1} r)r^{pn}}. $$

Let

$$\theta_{p,n}(r)=\frac{(1+\lambda_{p} r)a_{n}}{a_{2n}+a_{2n+1} r}. $$

Simple computation results in

$$\begin{aligned} \theta_{p,n}(r)&=\frac{ (1+\frac{1}{p}(1-\frac{1}{p}) r )}{1+\frac{(\frac{1}{p}+2n)(1-\frac{1}{p}+2n)}{(2n+1)^{2}}}\cdot\frac {a_{n}}{a_{2n}} \\ &\geqslant\frac{(1+\frac{1}{p}(1-\frac{1}{p}) r)}{1+\frac{(\frac {1}{p}+2n)(1-\frac{1}{p}+2n)}{(2n+1)^{2}}}\cdot\frac{a_{n}}{a_{2n}} \end{aligned}$$

by using Lemma 2.1.

(In fact, we easily know

$$\begin{aligned}& \frac{1}{p} \biggl(1-\frac{1}{p} \biggr)\leqslant\frac {1}{4} \leqslant\frac{(\frac{1}{p}+2n)(1-\frac{1}{p}+2n)}{(2n+1)^{2}} \\& \quad \Leftrightarrow\quad 4n^{2}+4n+1\leqslant4 \biggl( \frac{1}{p}+2n \biggr) \biggl(1-\frac{1}{p}+2n \biggr). \end{aligned}$$

Next, considering $1\leqslant p\leqslant2$, we only need to prove $12n^{2}+4n-1\leqslant0$. It is obvious.)

Setting

$$ Q_{p}(x)=\frac{\Gamma(\frac{1}{p}+x)\Gamma(1-\frac{1}{p}+x)\Gamma ^{2}(2x+1)}{\Gamma^{2}(x+1)\Gamma(\frac{1}{p}+2x)\Gamma(1-\frac{1}{p}+2x)}, $$

we have

$$\begin{aligned} \begin{aligned} \frac{Q'_{p}(x)}{Q_{p}(x)}={}&\psi \biggl(\frac{1}{p}+x \biggr)+\psi \biggl(1- \frac{1}{p}+x \biggr)+4\psi(2x+1)-2\psi(x+1) \\ &{}-2\psi \biggl(2x+ \frac{1}{2} \biggr)-2\psi \biggl(2x+1-\frac{1}{p} \biggr). \end{aligned} \end{aligned}$$

Using Lemma 2.2, we easily get

$$\begin{aligned} \frac{Q'_{p}(x)}{Q_{p}(x)} =&\psi \biggl(x+\frac{1}{p} \biggr)+\psi \biggl(x+1- \frac{1}{p} \biggr)+2\psi \biggl(x+\frac{1}{2} \biggr)-\psi \biggl(x+ \frac{1}{2p} \biggr) \\ &{}-\psi \biggl(x+\frac{1}{2}+\frac{1}{2p} \biggr)-\psi \biggl(x+ \frac{1}{2}-\frac{1}{2p} \biggr)-\psi \biggl(x+1-\frac {1}{2p} \biggr). \end{aligned}$$

Applying Lemma 2.2 again, we have

3.1 $$ \frac{1}{2} \biggl[\psi\biggl(x+\frac{1}{2p}\biggr)+ \psi\biggl(x+1-\frac{1}{2p}\biggr) \biggr]\leqslant\psi \biggl(x+ \frac{1}{2} \biggr). $$

Hence, we have

$$ \frac{Q'_{p}(x)}{Q_{p}(x)}>0. $$

It follows that the function $Q_{p}(x)$ is increasing in $x\in(0,\infty )$. So, we have

$$ \frac{a_{2n}}{a_{2n+1}}=Q_{p}(n)>Q_{p}(1)=\frac{4}{(\frac {1}{p}+1)(2-\frac{1}{p})} \geqslant\frac{16}{9}, $$

where we apply

$$ \biggl(\frac{1}{p}+1 \biggr) \biggl(2-\frac{1}{p} \biggr)\leqslant \biggl(\frac{\frac{1}{p}+1+2-\frac{1}{p}}{2} \biggr)^{2}= \biggl(\frac {3}{2} \biggr)^{2}=\frac{9}{4}. $$

Hence, we obtain

$$\begin{aligned} Q_{p,n}(r)&\geqslant\frac{16}{9}\frac{1+\frac{1}{p}(1-\frac {1}{p})}{1+\frac{(\frac{1}{p}+2n)(1-\frac{1}{p}+2n)}{(2n+1)^{2}}} \\ &\geqslant\frac{16}{9}\frac{1+\frac{1}{4}}{1+(\frac{4n+1}{4n+2})^{2}} \\ &=1+\frac{32n^{2}+104n+35}{9(32n^{2}+24n+5)} \\ &>1, \end{aligned}$$

and $f_{p}(r)>g_{p}(r)$.

On the other hand, since the function $K_{p}(r)$ is strictly increasing on $r\in(0,1)$, we have

$$ \frac{K_{p}(r)}{K_{p}(\sqrt[p]{r})}< 1. $$

Hence, we rewrite formula (1.3) as

$$u< u_{p}(r)=\frac{\frac{K_{p}(\sqrt[p]{r})}{K_{p}(r)}-1}{r}< \lambda. $$

Simple calculation leads to

$$u_{p}(r)=\frac{1}{r} \biggl(\frac{\sum_{n=0}^{\infty}\frac{(\frac {1}{p})_{n}(1-\frac{1}{p})_{n}}{(1)_{n}}\frac{r^{n}}{n!}}{\sum_{n=0}^{\infty }\frac{(\frac{1}{p})_{n}(1-\frac{1}{p})_{n}}{(1)_{n}}\frac {r^{pn}}{n!}}-1 \biggr) $$

and

$$\begin{aligned}& \lim_{r\rightarrow0}u_{p}(r)=\frac{1}{p}\biggl(1- \frac{1}{p}\biggr), \\& \lim_{r\rightarrow1}u_{p}(r)=0. \end{aligned}$$

The proof is complete.

## Conclusions

We show an elegant inequality involving the ratio of generalized complete elliptic integrals of the first kind and generalize an interesting result of Alzer.
